# A Case of Severe Acute Pancreatitis Following Endoscopic Biopsy of the Ampulla of Vater: A Rare Adverse Event of Esophagogastroduodenoscopy

**DOI:** 10.1002/deo2.70292

**Published:** 2026-01-28

**Authors:** Tetsushi Azami, Yuichi Takano, Go Akihiro, Mako Kitazono, Naoki Tamai, Jun Noda, Fumitaka Niiya, Kazuyuki Miyamoto, Masatsugu Nagahama

**Affiliations:** ^1^ Department of Medicine Division of Gastroenterology, Showa Medical University Fujigaoka Hospital Yokohama Japan; ^2^ Department of Emergency and Disaster Medicine Showa Medical University Fujigaoka Hospital Yokohama Japan

**Keywords:** acute pancreatitis, ampulla of vater, biopsy, endoscopic, postoperative complications

## Abstract

Histological biopsy is essential for diagnosing ampullary tumors; however, it can occasionally result in severe adverse events. A 49‐year‐old male underwent esophagogastroduodenoscopic screening, which revealed an ampulla of Vater with enlargement of the oral protrusion. An endoscopic biopsy was performed; several hours later, the patient developed severe acute pancreatitis requiring hospitalization. The biopsy result was benign, and no gallstones, ductal abnormalities, or other etiologies were identified on endoscopic ultrasonography or magnetic resonance cholangiopancreatography, and the biopsy was considered the most likely trigger. The patient recovered with conservative management and was discharged on day 14. No recurrence has been observed 3 months after discharge. Although acute pancreatitis following biopsy of the ampulla of Vater is extremely rare, it can be fatal. Endoscopists should be aware of this potential risk, carefully assess the necessity of biopsy, and ensure that patients provide informed consent before the procedure.

## 1 Introduction

Histological confirmation through biopsy is a critical component of diagnosis during esophagogastroduodenoscopy (EGD). Distinguishing between benign and malignant lesions of the ampulla of Vater based solely on endoscopic findings can be challenging, and a biopsy is often indispensable. According to the European Society of Gastrointestinal Endoscopy guidelines, an endoscopic biopsy is essential for the diagnosis of ampullary tumors [1].

Biopsy‐related adverse events (AEs) during EGD include bleeding and perforation [2]. A few cases of acute pancreatitis following ampullary biopsy have been reported, although such AEs are extremely rare and not well characterized [3, 4, 5, 6, 7]. We report a rare case of severe acute pancreatitis following endoscopic biopsy of a suspected ampullary lesion identified during EGD screening.

## 2 Case Report

**FIGURE 1:**
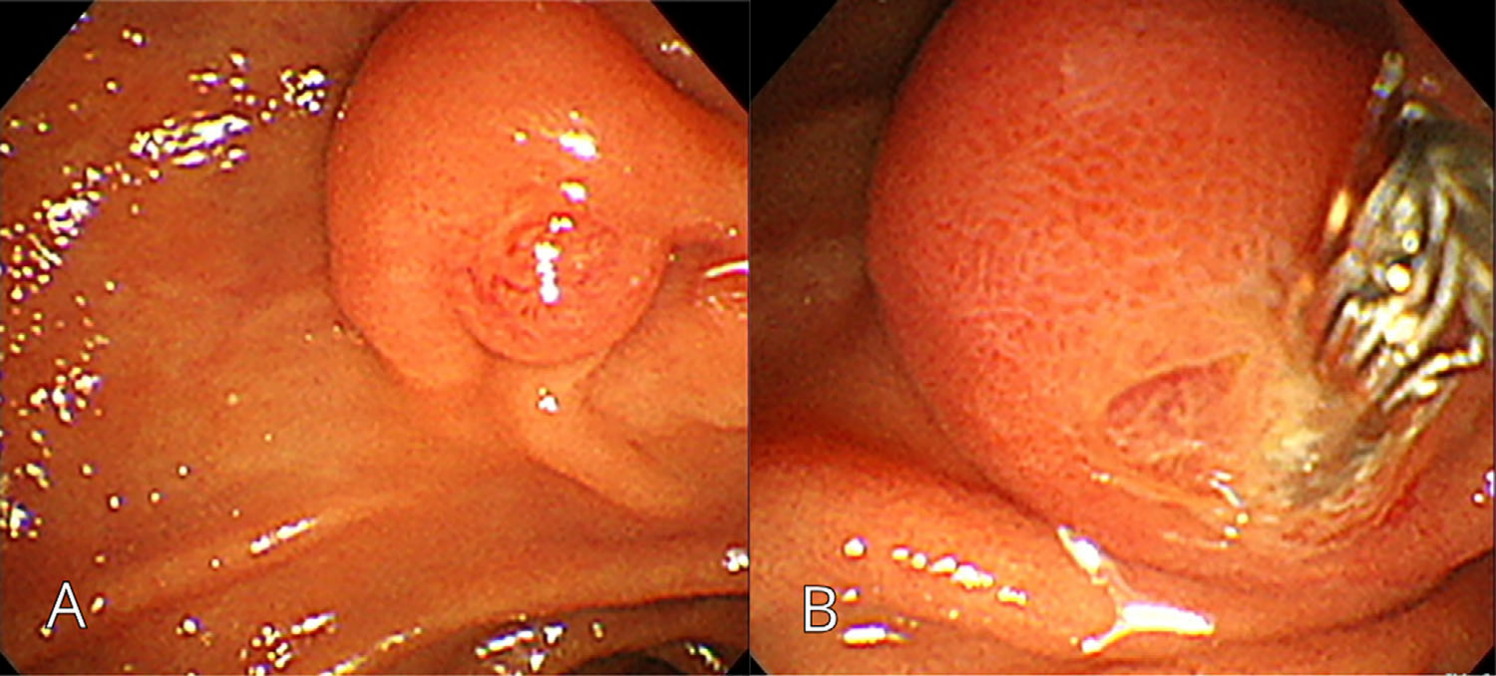
(A) Esophagogastroduodenoscopy reveals ampulla of Vater with enlargement of the oral protrusion. (B) Two biopsies were obtained from the ampulla of Vater by using a side‐viewing endoscope with oval‐cup biopsy forceps (Radial Jaw4P; Boston Scientific, Massachusetts, USA).

A 49‐year‐old male with a height of 175 cm, body weight of 64 kg, and a body mass index of 20.9 kg/m^2^ underwent EGD screening under sedation at a local hospital, during which an ampulla of Vater with enlargement of the oral protrusion was observed. The scope was changed to a side‐viewing duodenoscope, and two biopsy samples were obtained from the ampulla with oval‐cup biopsy forceps (Radial Jaw4P; Boston Scientific, Massachusetts, USA) during the same session (Figure 1). Although the biopsy was performed with the intention of avoiding the pancreatic duct orifice, retrospective review of the endoscopic images revealed that the biopsy site was located in close proximity to the pancreatic duct orifice. Hemostasis was confirmed, and the procedure was completed without any immediate AEs. However, a few hours after returning home, the patient developed upper abdominal pain and was admitted to the emergency department. He had no relevant medical history, had no history of alcohol or tobacco use, was not taking any medications, and had no relevant family medical history.

**FIGURE 2:**
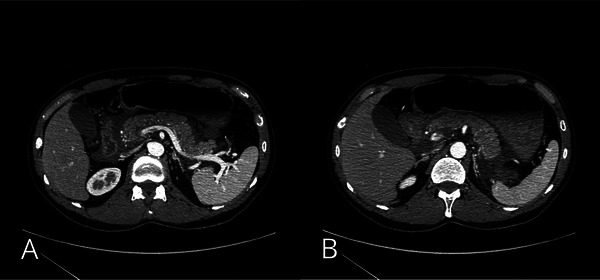
(A) Contrast‐enhanced computed tomography showing increased fat stranding around the pancreas. (B) The duodenum appears edematous, and gastric distension is observed.

Upon examination, the abdomen was flat and soft with spontaneous and tender pain localized in the epigastric region. Laboratory tests revealed elevated serum amylase levels of 2962 IU/L (reference range, 39–134 U/L). The white blood cell count was 6950 cells/µL, and C‐reactive protein was 0.04 mg/dL. Liver function test results were within normal ranges (23 U/L aspartate aminotransferase, 15 U/L alanine aminotransferase, 196 U/L alkaline phosphatase, and 1.3 mg/dL total bilirubin). Serum calcium (10.1 mg/dL), triglyceride (120 mg/dL), and immunoglobulin G4 (55 mg/dL) levels were within the normal limits, and renal function was preserved (0.96 mg/dL creatinine, 14.3 mg/dL blood urea nitrogen). Contrast‐enhanced computed tomography revealed increased fat stranding extending from the peripancreatic region to beyond the lower pole of the kidney. The duodenum was edematous, and the stomach was distended (Figure 2). A diagnosis of severe acute pancreatitis was made according to the Japanese clinical practice guidelines [8], based on a CT grade of 2 despite a prognostic factor score of 0. The patient was admitted for treatment with aggressive intravenous fluid resuscitation and continuous intravenous buprenorphine analgesia.

**FIGURE 3:**
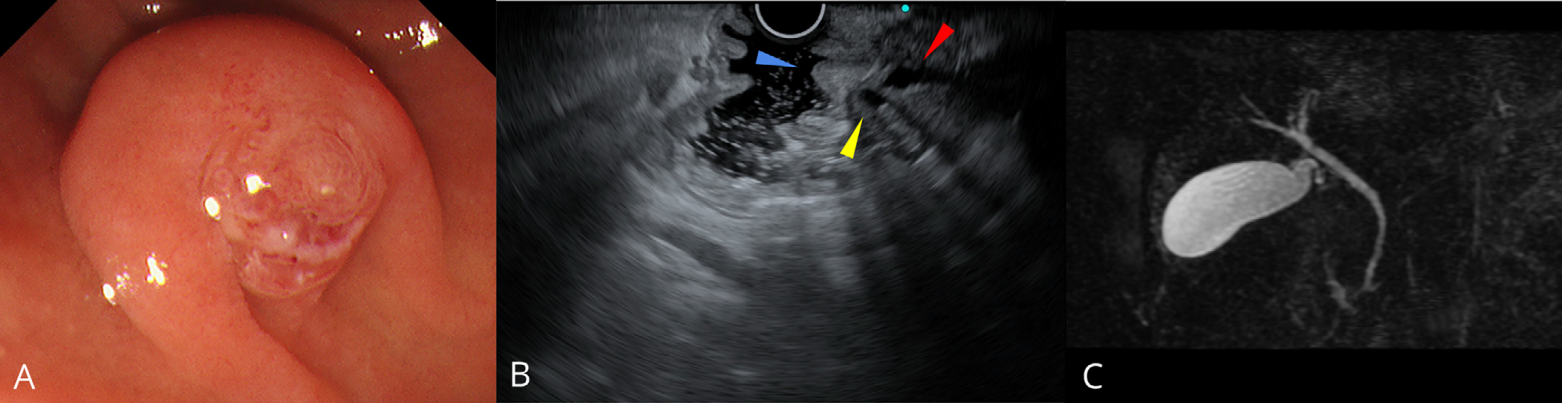
(A) Follow‐up esophagogastroduodenoscopy showing scarring at the biopsy site of the ampulla of Vater on day 4. (B) Endoscopic ultrasonography revealing no obvious abnormalities in the bile duct (red arrowhead), pancreatic duct (yellow arrowhead), or ampulla of Vater (blue arrowhead). (C) Magnetic resonance cholangiopancreatography revealing no abnormalities in the bile or pancreatic ducts.

EGD and endoscopic ultrasonography (EUS) were performed on day 4 of hospitalization. The duodenum appeared normal, and the biopsy site on the ampulla of Vater was scarred. EUS revealed no abnormalities in the biliary system or the ampullary mass. Magnetic resonance cholangiopancreatography revealed no biliary or pancreatic duct abnormalities (Figure 3). Transabdominal ultrasonography, EUS, and magnetic resonance imaging revealed no gallstones. There was no evidence of underlying diseases that could have triggered pancreatitis, as common etiologies were systematically excluded based on the medical history, laboratory findings, and imaging studies; therefore, based on the clinical course, the ampulla biopsy was considered the likely cause (Table 1).

The patient's clinical course was uneventful. Oral intake was resumed on day 3 without recurrence, and the patient was discharged on day 14. The biopsy specimen showed benign findings, including hyperplastic changes, inflammatory cell infiltration, and mild fibrosis. No recurrence has been observed 3 months after discharge.

## 3 Discussion

Common causes of acute pancreatitis include gallstones (40%), alcohol consumption (30%), hypertriglyceridemia (2%–7%), and drugs (< 5%), with other causes including hereditary factors, congenital anomalies, post‐endoscopic retrograde cholangiopancreatography (ERCP), trauma, infection, and malignancy [9]. In this case, all common causes of acute pancreatitis were excluded. Although rare etiologies and idiopathic pancreatitis cannot be completely ruled out, consideration of the clinical history and disease course suggested that the biopsy of the ampulla of Vater was the most likely triggering factor.

Rosella et al. evaluated biopsy‐related AEs of the ampulla of Vater by comparing groups with and without concurrent ERCP. Among the 154 patients who underwent biopsy with ERCP, five developed acute pancreatitis; none of the 92 patients who underwent biopsy alone developed acute pancreatitis [10]. To our knowledge, only five previous cases of acute pancreatitis caused solely by the biopsy of the ampulla of Vater have been reported [3, 4, 5, 6, 7], making this a rare occurrence.

The proposed mechanism for pancreatitis following biopsy of the ampulla of Vater includes obstruction of pancreatic juice outflow due to local edema or hematoma after biopsy [3]. This is likely attributable to the anatomical features of the ampulla of Vater. The common channel and the pancreatic duct within the papillary region are anatomically narrow. When papillary edema or minor intramural hemorrhage occurs after biopsy, this narrow outflow tract may become transiently obstructed, impairing pancreatic juice drainage. As a result, intraductal pressure may rise rapidly, leading to acute pancreatitis through an obstructive mechanism, even in the absence of direct pancreatic duct injury. In the present case, CT on admission revealed duodenal edema. Hemostasis was confirmed immediately after biopsy, and endoscopic observation in the early phase showed a scarred site, suggesting that edema rather than bleeding may have contributed to the ductal obstruction.

Ishida et al. identified patient‐related risk factors, including a small ampulla with a short common channel and a background of bleeding tendency [4]. Separately, Michopoulos et al. highlighted procedure‐related risk factors, noting that biopsy in close proximity to the pancreatic duct orifice may increase the risk of post‐biopsy pancreatitis. They also proposed that avoiding the pancreatic duct orifice during biopsy may reduce the risk of pancreatitis [6]. In our case, the biopsy was performed with a deliberate effort to avoid the pancreatic duct orifice, yet retrospective image review revealed that the sampling was taken closer to the pancreatic duct orifice than appreciated during the procedure (Figure 1). This indicates that precise avoidance of the pancreatic duct orifice may be challenging in some clinical settings, even when a side‐viewing duodenoscope is used.

Although no fatal cases have been reported, at least two of the five previous cases involved severe disease. One patient developed walled‐off necrosis (WON) requiring percutaneous necrosectomy and remained hospitalized for 168 days. Another patient also developed WON that required EUS‐guided drainage and was hospitalized for 137 days. Including the two severe cases, all previously reported cases described that the biopsy had been performed at a site distant from the pancreatic duct orifice, indicating that serious AEs may still occur even when the biopsy is technically appropriate. When incidental ampullary enlargement is observed, it may be prudent to postpone biopsy to a later date after a thorough explanation has been provided and informed consent has been obtained. Among the five previously reported cases of pancreatitis following ampullary biopsy, three cases were ultimately diagnosed as benign lesions, and the present case was also benign. Although the small number of reported cases precludes any meaningful evaluation of the association between histopathological diagnosis and the risk of pancreatitis, unnecessary biopsies should be avoided whenever possible. In this and all previously reported cases, pancreatitis developed on the same day as the biopsy. As early intervention improves outcomes in acute pancreatitis, patients undergoing a biopsy should be advised to seek immediate medical attention if they experience abdominal pain on the day of the procedure.

In conclusion, endoscopic biopsy of the ampulla of Vater is an important diagnostic tool; however, although rare, it carries the risk of severe acute pancreatitis. Endoscopists should be aware of this risk, carefully consider the necessity of biopsy, ensuring that patients receive adequate explanations and provide informed consent before the procedure.

## Author Contributions

**Conceptualization**: Tetsushi Azami. **Investigation**: Tetsushi Azami and Go Akihiro. **Supervision**: Yuichi Takano, Kazuyuki Miyamoto, and Masatsugu Nagahama. **Writing – original draft**: Tetsushi Azami. **Writing – review & editing**: Yuichi Takano, Go Akihiro, Mako Kitazono, Naoki Tamai, Jun Noda, Fumitaka Niiya, Kazuyuki Miyamoto, and Masatsugu Nagahama.

## Ethics Statement

The authors report the details of the patient's case in accordance with the ethical standards of the Helsinki Declaration of 1975, as revised in 2008(5).

## Funding

No funding was received for conducting this study.

## Conflicts of Interest

The authors declare no conflicts of interest.

4

**TABLE 1:** Summary of previously reported cases and the present case.

Reference	Publication year	Age (years)	Sex	Severity of pancreatitis	Endoscope type	EGD findings	Histopathology	Comorbidities	Treatment and procedures	Length of hospital stay	Clinical outcome
3	1994	40	M	N/A	Side‐viewing	Erythema	Adenoma	Gardner's syndrome, cranial osteomas, and an abdominal desmoid tumor	Conservative treatment	N/A	Survived
4	2013	53	M	Severe	Side‐viewing	Normal findings (prior tumor suspicion)	Benign	N/A	Conservative treatment, endoscopic ultrasonography‐guided drainage	137 days	Survived
5	2015	69	M	Severe	Forward‐viewing	Enlarged	Benign	Hypertension and osteoarthritis	Conservative treatment, mechanical ventilation, percutaneous necrosectomy	168 days	Survived
6	2016	51	M	N/A	Forward‐viewing	Mild granularity	Benign	Pulmonary embolism	N/A	Outpatient management	Survived
7	2023	59	M	N/A	Side‐viewing	Ulceration	Adenoma	None	Conservative treatment	2 days	Survived
Our case		49	M	severe	Side‐viewing	enlargement of the oral protrusion	Benign	None	Conservative treatment	14 days	survived

Abbreviations: EGD, esophagogastroduodenoscopy; EUS, endoscopic ultrasound; M, Male; N/A, not available or not reported; WON, walled‐off necrosis.

## Data Availability

The data supporting the findings of this case report are not publicly available due to patient privacy concerns, but are available from the corresponding author upon reasonable request.
